# Synthesis, Cyclopolymerization and Cyclo-Copolymerization of 9-(2-Diallylaminoethyl)adenine and Its Hydrochloride Salt

**DOI:** 10.3390/molecules171113290

**Published:** 2012-11-08

**Authors:** Kamal H. Bouhadir, Lara Abramian, Alaa Ezzeddine, Karyn Usher, Nikolay Vladimirov

**Affiliations:** 1 Department of Chemistry, American University of Beirut, Box 11-0236, Beirut, Lebanon; 2 Department of Chemistry, West Chester University, West Chester, PA 19383, USA; 3 Ashland Research Center, 500 Hercules Road, Wilmington, DE 19808, USA

**Keywords:** polynucleotide analogs, adenine, cyclopolymerization

## Abstract

We report herein the synthesis and characterization of 9-(2-diallylaminoethyl) adenine. We evaluated two different synthetic routes starting with adenine where the optimal route was achieved through coupling of 9-(2-chloroethyl)adenine with diallylamine. The cyclopolymerization and cyclo-copolymerization of 9-(2-diallylaminoethyl)adenine hydrochloride salt resulted in low molecular weight oligomers in low yields. In contrast, 9-(2-diallylaminoethyl)adenine failed to cyclopolymerize, however, it formed a copolymer with SO_2_ in relatively good yields. The molecular weights of the cyclopolymers were around 1,700–6,000 g/mol, as estimated by SEC. The cyclo-copolymer was stable up to 226 °C. To the best of our knowledge, this is the first example of a free-radical cyclo-copolymerization of a neutral alkyldiallylamine derivative with SO_2_. These polymers represent a novel class of carbocyclic polynucleotides.

## 1. Introduction

Novel methods for the preparation of modified oligodeoxynucleotides (ODNs) have been actively pursued in the last two decades [1,2,3] due to their potential use in therapeutic and diagnostic applications [4]. An important prerequisite of synthetic ODNs is their stability against biological nucleases that result in the cleavage of the phosphodiester backbone in RNA and DNA [5,6]. Extensive work has been conducted to modify or replace the phosphodiester backbone, furanose ring, nucleic base or a combination of two or more. A convenient route to form homopolymers resembling modified ODNs is through the cyclopolymerization of diallylamine derivatives thus replacing the phosphodiester and ribose moieties simultaneously. The cyclopolymerizations of diallyl quaternary ammonium salts have been thoroughly investigated during recent years [7,8]. The main interest in this research is the potential utility of the resulting polymers in industrial and pharmaceutical applications [9] such as layer-by-layer assembly [10,11,12], quantum dots, nanoparticle stabilization, paper industry [13], water treatment [14], metal electroplating, corrosion inhibition [15], cosmetic and hair treatments, antiperspirants, anion-exchange resins, antistatic agents, protein encapsulation, hydrogel formation [16], antibacterial properties [17,18] and drug delivery applications. The best studied of these compounds are the diallyldimethylammonium salts. In contrast, little work has been performed on the cyclopolymerization of the alkyldiallylammonium derivatives [19,20,21]. One route to prepare modified ODNs is based on the cyclopolymerization of quaternary diallylammonium salts with nucleic bases attached [22]. Poly(diallylquaternary ammonium salts) contain permanent positive groups that render them insoluble in nonpolar organic solvents and hence limit their utility in such applications. In contrast, polymers prepared from alkyldiallylammonium salts could be deprotonated to yield the corresponding neutral polymers. This characteristic of alkyldiallylammonium salts makes them attractive synthetic precursors in the preparation of neutral as well as cationic modified ODNs.

We report herein the synthesis and polymerization of 9-(2-diallylaminoethyl)adenine and its hydrochloride salt to form a new class of carbocyclic modified ODNs with ethylene or sulfone groups replacing the phosphodiester backbone and pyrrolidine rings replacing the furanose units of nucleic acids. 

## 2. Results and Discussion

**Scheme 1:**
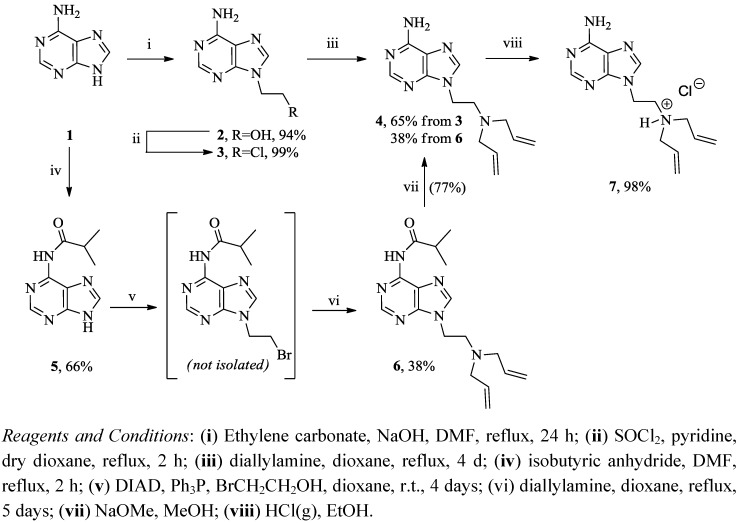
Synthesis of compounds **4** and **7**.

Two synthetic routes (Scheme 1) have been followed in the preparation of 1-(2-diallylaminoethyl)adenine [23]. In the first route, adenine was heated and maintained at reflux with ethylene carbonate in dry DMF to afford compound **2** that was chlorinated utilizing thionyl chloride in dry dioxane to yield the chloro derivative **3** [23,24]. Heating a mixture of **3** and an excess of diallylamine in dioxane at reflux conditions formed **4** in 60% overall yield from adenine [23]. Compound **7** was prepared in quantitative yield by passing HCl gas through a solution of **4** in anhydrous ethanol.

In an attempt to increase the overall yield of compound **4**, we evaluated an alternative synthetic route utilizing the Mitsunobu reaction (Scheme 1) [22,23,24]. Adenine was protected with isobutyric anhydride and coupled to bromoethanol *via* the Mitsunobu reaction with triphenylphosphine (Ph_3_P) and diisopropylazodicarboxylate (DIAD) to yield 6-isobutyryl-9-(2-bromoethyl)adenine (not isolated) that was heated and maintained at reflux with excess of diallylamine in dry dioxane to afford **6**. Hydrolysis of the isobutyryl group with sodium methoxide formed 9-(2-diallylaminoethyl)adenine (**4**) in 19% overall yield from adenine. These results clearly demonstrate the efficiency of the first route in comparison to the Mitsunobu route with more than three-fold increase in the overall yield.

**Scheme 2:**
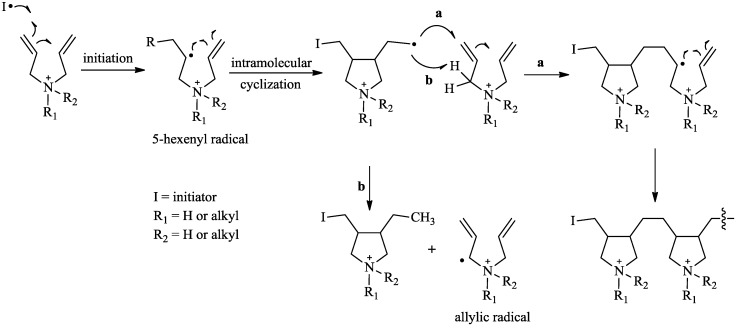
Mechanism of the cyclopolymerization of quaternary diallylammonium salts.

The cyclopolymerization and cyclo-copolymerization of diallylammonium chloride (DAAC) have been reported to yield poly(diallylammonium chloride) (PDAAC) and poly(diallylammonium chloride-co-sulfur dioxide) (PDAAC-SO_2_), respectively [25,26,27]. Homopolymerizations of methacrylate-type monomers containing nucleic acid bases have also been reported to yield modified nucleosides [22,28,29,30,31]. This compelled us to investigate the homopolymerization and copolymerization of monomers **4** and **7** with SO_2_. The pioneering work of Butler and co-workers has set the foundations for the cyclopolymerization of diallyl monomers [32]. The presently accepted mechanism for the cyclopolymerization of diallylammonium salts is depicted in scheme 2 [33]. The initiator attacks C-1 forming the 5-hexenyl radical that cyclizes *via* the favored 5-exo-trig mode to yield a highly reactive and nucleophilic primary radical (Scheme 2). The primary radical attacks another monomer (intermolecular propagation pathway a) or abstract an allylic hydrogen (degradative chain transfer pathway b) to yield a stable allylic radical. 

**Scheme 3:**
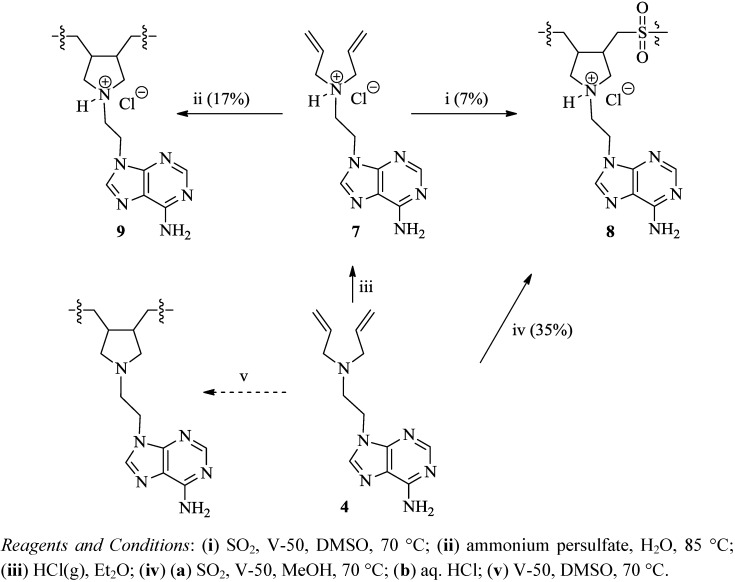
Cyclopolymerization of compounds **4** and **7**.

The cyclopolymerization of monomer **7** was initiated with ammonium persulfate, *tert-*butyl hydro-peroxide or V-50 initiator (Scheme 3). Compound **7** was cyclopolymerized with ammonium persulfate in water to yield polymer **9** in 17% yield whereas lower yields of 5% and 3.7% were obtained with V-50 and *tert-*butyl hydroperoxide respectively (Table 1). In contrast, the cyclopolymerization of DAAC in our laboratory was more efficient using V-50 in water to yield PDAAC in 67% yield. The low polymerization efficiency of monomer **7** in this study is consistent with reported cyclopolymerizations of related alkyldiallylammonium chlorides [34]. This difficulty in the polymerization is attributed to an increase in the rate of the degradative chain transfer reaction (pathway b, Scheme 2) through the abstraction of the α-hydrogen from the allylic position of the monomer resulting in a stable allylic radical [35]. A similar conclusion was derived for the relatively low degree of cyclopolymerization of monomer **4** (Entry 11, Table 1) since neutral diallyl monomers are known to have a more effective degradative chain transfer [36,37] than their charged counterparts.

**Table 1:** Reaction conditions for the cyclopolymerization of **DAAC**, **7** and **4** ^a^.

Entry	Initiator ^b^	Monomer	Solvent	Monomer	T	Yield	Mn ^d^	Mw	Mz	PDI	dn/dc	
		1		2	(°C)	(%) ^c^	(kDa)	(kDa)	(kDa)			
											(mL/g)	
1	V-50	DAAC	H_2_O	-	70	67	14.1	28.8	54.5	1.98	0.173	
2	TBHP	7	H_2_O	-	80	3.7	-	-	-	-	-	
3	V-50	7	H_2_O	-	70	5	-	-	-	-	-	
4	APS	7	H_2_O	-	85	17	2	2.5	3	1.25	0.183	
5	V-50	DAAC	MeOH	SO_2_	70	92	1350	1700	2190	1.32	0.181	
6	V-50	7	MeOH	SO_2_	70	trace	-	-	-	-	-	
7	V-50	7	DMSO	SO_2_	70	7	-	-	-	-	-	
8	V-50	**4**	MeOH	SO_2_	70	35	6	6.2	6.3	1.03	0.183	
9	VPE-0201	**4**	MeOH	SO_2_	70	10.6	-	-	-	-	-	
10	V-50	**4**	DMSO	SO_2_	70	trace	-	-	-	-	-	
11	V-50	**4**	DMSO	-	70	trace	-	-	-	-	-	

^a^ All reactions were conducted in sealed tubes after degassing with nitrogen gas for 10 min (for aqueous solutions) or freeze-thaw degased (3 cycles) for methanol and DMSO. Reactions were heated for 3 days; ^b^ V-50: 2,2'-azobis(2-methylpropionamidine)dihydrochloride, TBHP: *tert*-butyl hydroperoxide, APS: ammonium persulfate; VPE-0210: macro azo initiator; ^c^ Isolated yield after dialysis (for aqueous solutions) or after trituration in methanol followed by filtration (3 cycles); ^d^ The separations were carried out on a PSS Novema pre-column connected in series to three PSS Novema columns (30 Å, 1000 Å, 10,000 Å). Aqueous oxalic acid (0.22 M) was used as the mobile phase at 40 °C with nominal flow rate of 0.8 mL/min.

**Scheme 4:**
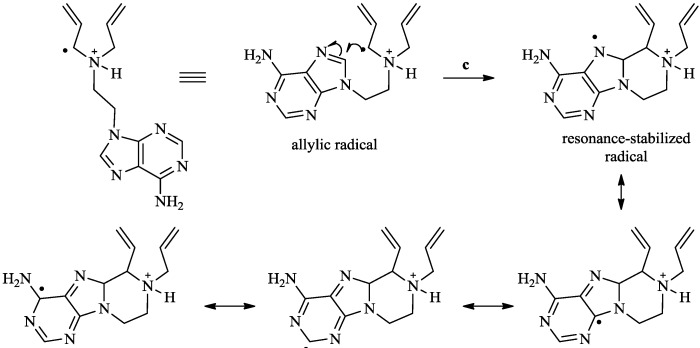
Cyclization of the allylic radical.

The copolymerizations of olefins with SO_2_ typically result in high molecular weight polymers in good yields [38]. This is due to the introduction of a flexible sulfonyl radical into the propagating polymer chain that reduces its rigidity and increases its solubility [39]. The cyclo-copolymerization of DAAC with SO_2_ was initiated with V-50 in methanol to result in the formation of PDAAC-SO_2_ in high yields (Entry 5, Table 1). In contrast, compound **7** underwent cyclo-copolymerization with SO_2_ when initiated with V-50 in DMSO to form polymer **8** in 7% yield, whereas only traces of the polymer were detected when the reaction was conducted in methanol (Entries 6 and 7, Table 1). The yield of the copolymerization of **8** was lower than usual, which suggests that the rate of the degradative chain transfer (pathway b, Scheme 2) has increased further in this case. This increase may be ascribed to the cyclization of the allylic radical (Scheme 4) through an intermolecular attack on the C-8 carbon of the adenine ring to form a thermodynamically stable 6-membered ring as well as a resonance-stabilized radical specie. A relatively high yield was achieved for the copolymerization of the neutral monomer **4** and SO_2_ to form polymer **8**. V-50 was the optimal initiator resulting in a 35% yield in comparison to 10.6% with the macroinitiator VPE-0201 (Entries 8 and 9, Table 1). The copolymerization of neutral allylated monomers with SO_2_ have been recently reported to yield low molecular weight oligomers in good yields [40,41]. The polymerization efficiency was attributed to the formation of a complex between SO_2_ and the diallyl groups of the monomer. This complex presumably facilitates the addition of the primary radical to SO_2_ forming a stable sulfonyl radical which attacks an unreacted monomer to yield a propagating polymer chain.

**Figure 1:**
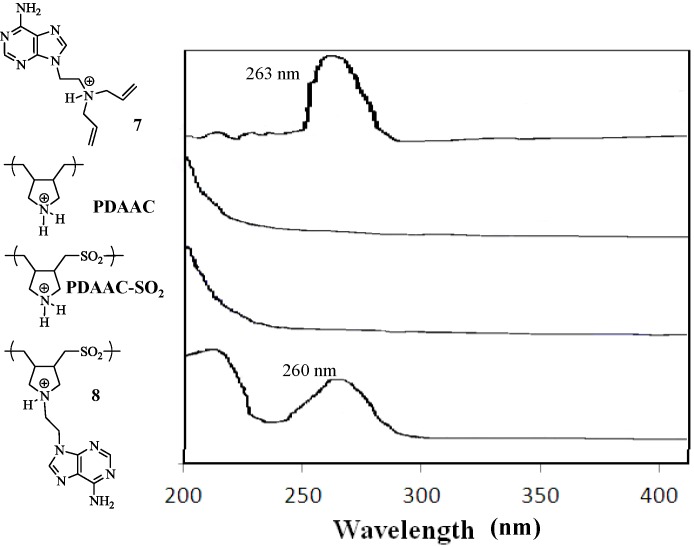
UV-VIS spectra of **7**, **PDAAC**, **PDAAC-SO_2_** and **8**.

**Figure 2:**
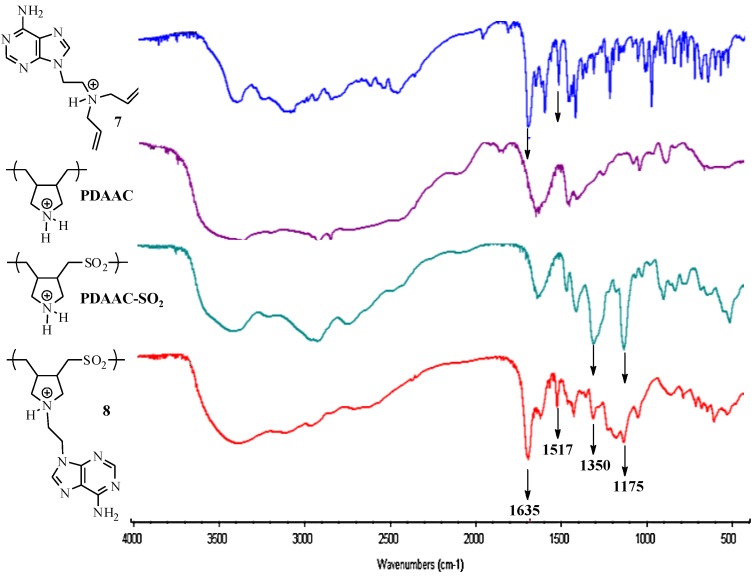
FTIR spectra of **7**, **PDAAC**, **PDAAC-SO_2_** and **8**.

We evaluated the solubility of PDAAC-SO_2_ and **8** in a variety of solvents as seen in Table 2. Both polymers, PDAAC-SO_2_ and **8**, displayed similar solubility in most solvents investigated, except DMSO, where **8** was soluble at room temperature and PDAAC-SO_2_ was not soluble at 2 wt%. This difference in solubility could be attributed to the hydrophobic nature of the purine ring in **8**. The UV-VIS absorption spectra display a minor shift in the λ_max_ of compound **7** from 263 nm to 260 nm for polymer **8** (Figure 1). This absorption is absence in polymers PDAAC and PDAAC-SO_2_ due to the absence of the purine ring. The FTIR spectrum of **8** reveals a signal at 1691 cm^−1^ that is present in the corresponding monomer **7** (Figure 2) while the spectra of PDAAC and PDAAC-SO_2_ show only the presence of a broad peak at 1635 cm^−1^. In addition, FTIR revealed the presence of two peaks at 1350 cm^−1^ and 1157 cm^−1^ assigned to the symmetric and asymmetric vibrations of the sulfone groups in both PDAAC and PDAAC-SO_2_. 

**Table 2:** Solubility (2% w/w) of **PDAAC-SO_2_** and **8** in selected solvents.

			PDAAC-SO_2_		8	
Solvent	Є ^a^	bp (°C)	Cold ^b^	Hot ^c^	Cold ^b^	Hot ^c^
Formamide	111	210	+	+	+	+
Water	78.4	100	+	+	+	+
Formic Acid	58.5	100–101	+	+	+	+
DMSO	47.2	189	+	±	−	±
DMF	38.3	153	−	−	−	−
Ethylene glycol	37.3	196–198	±	+	±	+
Methanol	32.3	65	−	−	−	−
Ethanol	24.3	78	−	−	−	−
Acetone	20.7	56	−	−	−	−
Diglyme	7	162	−	−	−	−
Dioxane	2.2	101	−	−	−	−

^a^Є: dielectric constant; ^b^ at room temperature; ^c^ at the boiling point; +: soluble; −: insoluble; ±: partially soluble.

**Figure 3:**
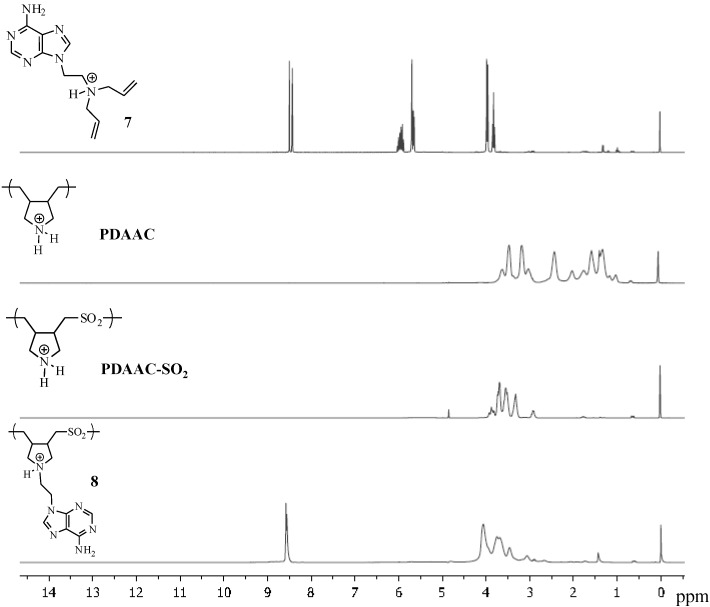
^1^H-NMR spectra of **7**, **PDAAC**, **PDAAC-SO_2_** and **8**.

**Figure 4:**
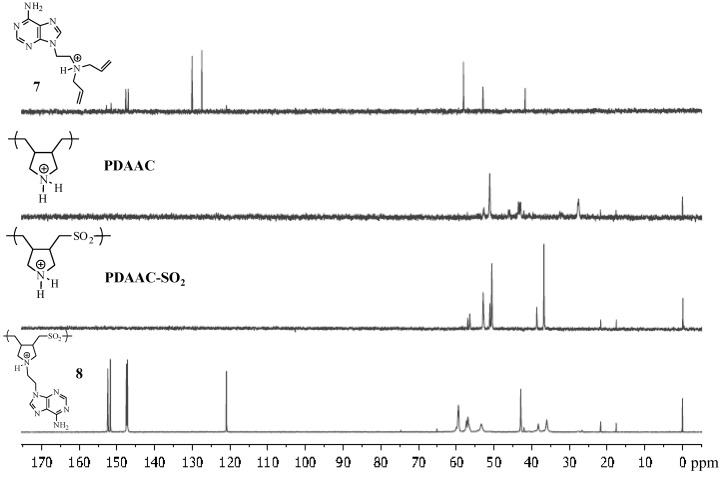
^13^C-NMR spectra of **7**, **PDAAC**, **PDAAC-SO_2_** and **8**.

The ^1^H-NMR spectrum of compound **8** (Figure 3) showed the aromatic C-H signals of the purine at 8.47 and 8.49 ppm in comparison to the same signals at 8.3 and 7.9 ppm in the corresponding monomer **7**. In addition, the vinylic protons at 6 and 5.6 ppm for the monomer have disappeared in polymer **8**. A similar trend was seen in the ^13^C-NMR (Figure 4) where the signals of the purine ring are seen at 151, 152.2, 147.5, 147.2 and 120.7 ppm. In addition, the signal for the vinylic carbons in the 130–140 ppm region of **7** disappeared in polymer **8**.

**Figure 5:**
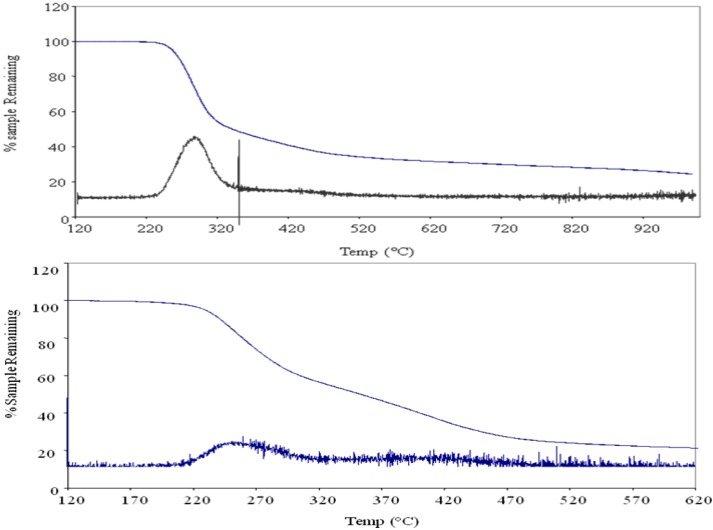
TGA thermograms for the thermal degradation of **PDAAC-SO_2_** (top) and **8** (bottom) at a heating rate of 10 °C min^−1^ under a flow of nitrogen gas.

**Figure 6:**
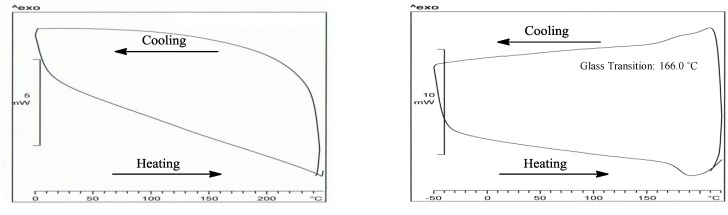
DSC thermograms of **PDAAC-SO_2_** (**left**) and **8** (**right**) for the first cooling cycle and second heating cycle at a heating rate of 10 °C min^−1^ under a flow of nitrogen gas.

Thermogravimetric analysis (TGA) and Differential Scanning Calorimetry (DSC) were used for the thermal analysis of the cyclo-copolymers. The TGA thermograms are shown in Figure 5. The TGA of polymer **8** exhibited an onset temperature of 226 °C with dm/dTmax = 261 °C whereas PDAAC-SO_2_ has an onset temperature of 251 °C with a dm/dTmax = 287 °C. Copolymer **8** was thermally stable up to a temperature of 226 °C below that of PDAAC-SO_2_. Around 79% of the polymer weight was lost during the first transition for **8** in comparison to only 49% weight loss for PDAAC-SO_2_. The weight loss for PDAAC-SO_2_ is attributed to depolymerization and release of HCl and SO_2_. In contrast, the degradation of **8** is initiated at a lower temperature presumably due to the breakdown of the thermally sensitive adenine moiety. Figure 6 shows the DSC thermograms of PDAAC-SO_2_ and polymer **8**. No discernible peaks are found for PDAAC-SO_2_ in the temperature range of 0–240 °C. In contrast, a distinct glass transition state is seen for **8** at 166 °C. The molecular weight distributions of polymers **8** and **9** are shown in Table 1. The average molecular weight of PDAAC was 28.8 kDa whereas the copolymer PDAAC-SO_2_ was around 1,700 kDa (Entry 1, Table 1). This is consistent with reported increase in molecular weight distributions of related copolymers with SO_2_. A similar trend (but to a lesser extent) was observed for the cyclopolymerization of **4** and **7** that resulted in a 2-fold increase in the molecular weight of copolymer **8** in comparison to **9**. 

## 3. Experimental

### 3.1. General

Reagents used in the syntheses were purchased from the Aldrich Chemical Company (Milwaukee, WI, USA), ACROS Chemicals (Loughborough, UK), Fisher Scientific Company (Fair Lawn, NJ, USA) and were used as received. Dioxane was dried over sodium metal and distilled directly before use. Disposable dialysers (MWCO 1000 Da), cellulose acetate/cellulose nitrate mixed esters membranes, were purchased from the Sigma Aldrich Chemical Company (Steinheim, Germany). Slide-A-Lyzer 3.5K dialysis cassettes (MWCO 3500) were purchased from Pierce (USA). The macro azo initiators VPE-0201 and the water-soluble initiator 2,2'-azobis(2-methylpropionamidine)dihydrochloride (V-50) were obtained from Wako Pure Chemical Industries (Richmond, VA, USA) and were used as received. 

### 3.2. Measurements

Melting points were determined on a Mettler Toledo FP62 apparatus and are uncorrected. NMR spectra were determined in deuterated solvents with tetramethylsilane (TMS) or sodium 2,2,-dimethyl-2-silapentane-5-sulfonate (DSS) as the internal standards on a Bruker AV 300 NMR spectrometer. Chemical shifts are reported in ppm (δ) downfield relative to TMS or DSS. Infrared spectra were recorded as KBr pellets using a Nicolet 4700 FTIR spectrometer with a Hewlett Packard Desk jet 840C plotter. The IR bands are reported in wave numbers (cm^−1^). The UV-VIS absorption spectrum was measured using Jasco V-570 UV/VIS/NIR spectrometer. SEC analysis was performed on a liquid chromatograph consisting of a Waters Breeze solvent delivery system and Waters M717 autosampler (Waters Corporation, Milford, MA, USA), a DAWN EOS light scattering photometer and an OPTILAB rEX differential refractive index detector (Wyatt Technology Corporation, Santa Barbara, CA, USA). Aqueous oxalic acid (0.22 M) at 40 °C with nominal flow rate of 0.8 mL/min was used as the mobile phase. The separations were carried out on a PSS Novema pre-column connected in series to three PSS Novema columns (30 Å, 1,000 Å, 10,000 Å) from Polymer Standard Service (Amherst, MA, USA) (8.0 mm × 300 mm, 10 μm). All samples were prepared by stirring overnight in the mobile phase at a concentration of 1–2 mg/mL and filtered through 0.45 μm PVDF membrane filter. The TGA data was obtained using TGA2050 Thermogravimetric Analyzer (TA Instruments, New Castle, DE, USA) and Thermal Advantage software, v1.1A. The samples were equilibrated at 120 °C and the temperature was increased to 1,000 °C at a rate of 10 °C/min under a flow of nitrogen gas. The DSC data was obtained using a Mettler Toledo DSC821 and Star Software v 8.10. The samples were weighed directly into an aluminium pan with lid using a microbalance. Samples were preheated to 220 °C, cooled to −50 °C at a rate of −20 °C/min then heated to 220 °C at a rate of 20 °C /min.

*9-(2-Hydroxyethyl)adenine* (**2**). A one liter round-bottom flask was charged with adenine (14.9 g, 0.11 mol), ethylene carbonate (10.6 g, 0.12 mol), sodium hydroxide (0.5 g, 12.5 mmol), and dry DMF (450 mL) and the mixture was heated and maintained at reflux overnight. The solvent was removed under reduced pressure and the residue was crystallized from ethanol to yield a white solid (19.71 g, 94%). Mp 238–239 °C; ^1^H-NMR (300 MHz, DMSO-d_6_) δ 3.5 (t, 2H, *J* = 5.45 Hz), 4.0 (t, 2H, *J* = 5.32 Hz), 5.1 (br s, 1H), 7.0 (br s, 2H), 7.9 (s, 1H), 7.95 (s, 1H); ^13^C-NMR (75 MHz, DMSO-d_6_) δ 45.6 (CH_2_), 59.1 (CH_2_), 118.6 (C), 141.3 (CH), 149.5 (CH), 152.2 (C), 155.8 (C); UV-VIS λ_max_ 265 nm; IR (KBr) 3311, 3246, 3056, 2930, 2865, 1687, 1607, 1574, 1067, 1018 cm^−1^. Spectroscopic data are consistent with those reported in the literature [23,24,25].

*9-(2-Chloroethyl)adenine* (**3**). A 250 mL round-bottom flask was charged with **2** (10.63 g, 59.3 mmol), dry dioxane (330 mL), freshly distilled pyridine (4 mL), and thionyl chloride (83 mL, 114 mmol). The reaction mixture was heated and maintained at reflux for two hours. The solvent was removed under reduced pressure and the residue was triturated with carbon tetrachloride, filtered, and the solid was recrystallized from ethanol (11.6 g, 99%). Mp 204–205 °C; ^1^H-NMR (300 MHz, D_2_O) δ 4.0 (t, 2H, *J* = 5.71 Hz), 4.7 (t, 2H, *J* = 5.72 Hz), 8.1 (s, 1H), 8.4 (s, 1H); ^13^C-NMR (75 MHz, D_2_O) δ 45.4 (CH_2_), 48.7 (CH_2_), 120.9 (C), 147.2 (CH), 147.8 (CH), 151.3 (C), 152.7 (C); ^1^H-NMR (300 MHz, DMSO-d_6_) δ 3.75 (t, 2H, *J* = 5.43), 4.2 (t, 2H, *J* = 5.43 ), 8.1 (s, 1H), 8.15 (s, 1H); ^13^C-NMR (75 MHz, DMSO-d_6_) δ 42.9 (CH_2_), 45.3 (CH_2_), 117.8 (C), 144.1 (CH), 145.0 (CH), 148.5 (C), 150.2 (C); UV-VIS λ_max_ 261 nm; IR (KBr) 3342, 3062, 2083, 1701, 1614, 1559, 1518, 1411 cm^−1^. Spectroscopic data are consistent with those reported in the literature [23,24,25].

*9-(2-Diallylaminoethyl)adenine* (**4**). (From compound 3): A 100 mL round bottom flask was charged with **3** (5 g, 25.3 mmol), dry dioxane (100 mL) and diallylamine (14.7 g, 151.8 mmol). The reaction mixture was heated and maintained at reflux for four days. The solvent was removed under reduced pressure to yield a solid residue that was dissolved in aqueous HCl (10%, 30 mL), washed with CHCl_3_ (4 × 40 mL), rendered basic with sodium hydroxide pellets, and extracted with CHCl_3_ (4 × 40 mL). The organic layers were combined, dried over anhydrous magnesium sulfate, filtered and the solvent was removed under reduced pressure. The residue was crystallized from acetone-hexane and dried under reduced pressure to yield a pale beige powder (4.26 g, 65%). Mp 142–144 °C; ^1^H-NMR (300 MHz, CDCl_3_) 2.80 (t, 2H, *J* = 5.9 Hz), 3.05 (d, 2H, *J* = 6.4 Hz), 4.18 (d, 1H, *J* = 5.8 Hz), 5.04 (m, 2H), 5.60 (m, 1H), 5.90 (br s, 2H), δ 7.85 (s, 1H), 8.28 (s, 1H); ^13^C-NMR (75 MHz, CDCl_3_) 57.1 (CH_2_), 41.1 (CH_2_), 52.1 (CH_2_), δ 118.1 (CH_2_), 119.3 (C), 134.7 (CH), 141.4 (CH), 150.0 (C), 152.6 (CH), 155.3 (C); UV-VIS λ_max_ 264 nm; IR (KBr) 3290, 3119, 2979, 2801, 2679, 1670, 1644, 1602, 1574, 1514, 1478, 1414, 1352, 1322, 1205, 909 cm^−1^. HRMS (EI) calcd for C_13_H_18_N_6_ (M^+^), 258.15929, found 258.15860.

*9-(2-Diallylaminoethyl)adenine* (**4**). (From compound 6): A 100 mL three-necked round-bottom flask immersed in an ice-water bath was charged with methanol (10 mL) and sodium (0.1 g, 4.3 mmol). A solution of **6** (0.5 g, 1.52 mmol) in methanol (10 mL) was added drop-wise under a flow of nitrogen gas. The ice-water bath was removed and the mixture was heated and maintained at reflux for four hours then stirred overnight at ambient temperature. The reaction flask was immersed in an ice-water bath and distilled water (10 mL) was added. The volume was then reduced to 1/10 of its initial volume under reduced pressure. The mixture was acidified with aqueous HCl (10%, 5 mL) and washed with chloroform (3 × 20 mL). The aqueous layer was separated, neutralized with aqueous NaOH (10%, 5 mL) and the product was extracted with chloroform (3 × 25 mL). The organic layers were combined, dried over anhydrous sodium sulfate and filtered. The solvent was removed under reduced pressure to yield a pale yellow solid (0.3 g, 77%).

*N^6^-Isobutyryladenine* (**5**). A 50 mL round-bottom flask was charged with adenine (1 g, 7.4 mmol), isobutyric anhydride (3.617 g, 22.2 mmol), and dry DMF (25 mL). The mixture was heated and maintained at reflux for two hours resulting in a clear yellow solution. The solvent was removed under reduced pressure and the crude solid was crystallized from a mixture of ethanol and water (30 mL, 1:1) to yield white crystals (1 g, 66%). Mp 228–230 °C; ^1^H-NMR (300 MHz, DMSO-d_6_) δ 1.18 (d, 6H, *J* = 6.8 Hz,), 2.96 (hep, 1H, *J* = 6.8 Hz), 8.40 (s, 1H), 8.60 (s, 1H); ^13^C-NMR (75 MHz, DMSO-d_6_) δ 19.1 (CH_3_), 34.0 (CH), 113.6 (C), 144.3 (C), 145.5 (CH), 151.1 (CH), 161.3 (C), 177.0 (C); IR (KBr) 3280, 3068, 2974, 2825, 1687, 1655, 1626, 1554, 1513, 1466, 1432, 1390, 1369, 1328, 1307, 1219 cm^−1^. Spectroscopic data are consistent with those reported in the literature [22,23,24,25].

*N^6^-Isobutyryl-9-(2-diallylaminoethyl)adenine* (**6**). A 250 mL three-necked round-bottom flask was charged with compound **5** (0.5 g, 2.43 mmol), bromoethanol (0.375 g, 2.92 mmol), triphenyl phosphine (1.286 g, 4.8 mmol), and dry dioxane (100 mL). The flask was partially immersed in an ice-water bath and a solution of diisopropylazodicarboxylate (1.039 g, 4.8 mmol) in dry dioxane (50 mL) was added drop-wise under an atmosphere of nitrogen. The solution turned clear halfway through the addition. The ice-water bath was removed and the reaction was stirred at room temperature for four days under nitrogen. The solid precipitate was removed by filtration and the filtrate was evaporated under reduced pressure. Diallylamine (0.487 g, 4.86 mmol), and dry dioxane (50 mL) were added to the oily residue and the mixture was heated and maintained at reflux for five days. The solvent was evaporated under reduced pressure and the residue was acidified with aqueous HCl (10%, 10 mL), washed with dichloromethane (3 × 10 mL), neutralized with aqueous NaOH (10%, 10 mL), and extracted with dichloromethane (3 × 20 mL). The organic layers were combined, dried over anhydrous sodium sulfate and filtered. The solvent was removed under reduced pressure and the oily residue was triturated with hexane. The white solid was filtered and dried under reduced pressure to yield the product (0.3 g,37.5%). Mp 88–90 °C; ^1^H-NMR (300 MHz, CDCl_3_) δ 1.2 (d, 6H, *J* = 6.8 Hz), 2.8 (d, 1H, *J* = 5.7 Hz), 2.8 (d, 1H, *J* = 5.8 Hz), 3.1 (d, 2H, *J* = 6.3 Hz), 3.2 (hep, 1H, *J* = 6.8 Hz), 4.3 (d, 1H, *J* = 5.5 Hz), 4.3 (d, 1H, *J* = 6.0 Hz), 3.1 (d, 2H, *J* = 6.3 Hz), 5.1(m, 2H), 5.6 (m, 1H), 8.2 (s, 1H), 8.7 (s, 1H), 10.0 (br s, 1H); ^13^C-NMR (75 MHz, CDCl_3_) δ 117.8 (CH_2_), 56.9 (CH_2_), 134.7 (CH), 42.1 (CH_2_), 51.8 (CH_2_), 151.6 (C), 143.7 (CH), 121.9 (C), 149.3 (C), 152.0 (CH), 176.6 (C), 35.5 (CH), 19.1 (CH_3_); IR (KBr) 3544, 3304, 3172, 3090, 3034, 2970, 2925, 2806, 1709, 1675, 1611, 1579, 1542, 1489, 1458, 1436, 1401, 1349, 1316, 1275, 1216, 921 cm^−1^. HRMS (ESI) calcd for C_17_H_25_N_6_O (M+1)^+^, 329.208436, found 329.2091.

*9-(2-Diallylaminoethyl)adenine.HCl* (**7**). Dry HCl gas, generated by the drop-wise addition of concentrated H_2_SO_4_ to NaCl, was bubbled through a clear solution of 9-(2-diallylaminoethyl)adenine (3.5 g, 13.5 mmol) in ethanol (400 mL). The solid precipitate was filtered, washed with ethanol and dried under reduced pressure (3.91 g, 98%); Mp 202–204 °C; ^1^H-NMR (300 MHz, D_2_O) δ 3.8 (t, 2H, *J* = 6.62 Hz), 3.9 (d, 4H, *J* = 7.27 Hz), 4.9 (t, 2H, *J* = 6.62 Hz), 5.6 (d, 4H, *J* = 5.65 Hz), 5.7 (s, 1H), 5.9 (m, 2H, *J* = 5.92 Hz), 8.4 (s, 1H), 8.47 (s, 1H); ^13^C-NMR (75 MHz, D_2_O) δ 41.8 (CH_2_), 53.1 (CH_2_), 58.2 (CH_2_), 121.1(C), 127.6 (CH_2_), 130.2 (CH), 147.1 (CH), 147.7 (CH), 151.6 (C), 152.9 (C); UV-VIS λ_max_ 263 nm; IR (KBr) 3396, 3080, 2980, 2939, 1691, 1616, 1649, 1595, 1514, 1449, 1436, 1416, 1214, 971 cm^−1^; GC: I = 949, retention time 18 min; MS (EI) *m/z* (relative intensity) 258.2 (M−HCl, 0.05), 217 (32), 136 (25), 110 (100), 108 (21), 41 (57); HRMS (EI) m/e calcd for C_13_H_18_N_6_ (M−HCl)^+^ 258.15929, found 258.16146.

### 3.3. Procedure for Cyclopolymerization of DAAC with V-50

A glass tube (8 mm i.d., 11 mm o.d.) was charged with DAAC (2 g, 14.96 mmol) and distilled water (8 mL) and placed in a preheated oil bath at 70 °C. The tube was sealed with a septum and the mixture was purged with nitrogen gas for twenty minutes. V-50 (30 mg, 0.11 mmol) was added and the mixture was purged with nitrogen gas for an additional ten minutes, stirred and heated at 70 °C for three days. The polymer was precipitated with the addition of EtOH. The gelatinous solid was dried under reduced pressure. The solid was triturated in EtOH, filtered and dried under reduced pressure (1.35 g, 67%): M.p. decomposed at 305 °C; ^1^H-NMR (300 MHz, D_2_O) δ 0.9 (br s), 1.1 (br s), 1.3 (br s), 1.5 (br s), 1.6 (br s), 1.9 (br s), 2.3 (br s), 2.9 (br s), 3.1 (br s), 3.4 (br s), 3.5 (br s); ^13^C-NMR (75 MHz, D_2_O) δ 26.9 (CH_2_), 31.6 (CH_2_), 42.6 (CH), 45.2 (CH), 50.7 (CH_2_), 52.2 (CH_2_); FTIR 3363, 2922, 1635, 1456 cm^−1^.

### 3.4. Procedure for Cyclo-Copolymerization of DAAC with V-50

A glass tube (8 mm i.d., 11 mm o.d.) was charged with DAAC ( 1 g, 7.48 mmol), a solution of MeOH (1.65 mL) containing SO_2_ (0.48 g, 7.48 mmol) and V-50 (15 mg, 55.3 μmol). The mixture was freeze-thawed degassed (three cycles) and the tube was sealed under reduced pressure. The mixture was heated at 65–70 °C for three days. The white precipitate was triturated with MeOH, filtered and dried under reduced pressure to yield a white solid (1.36 g, 92%): M.p. decomposed above 240 °C; ^1^H-NMR (300 MHz, D_2_O) δ 3.3 (br s), 3.5 (br s), 3.6 (br s), 3.8 (br s); ^13^C-NMR (75 MHz, D_2_O) δ 36.8 (CH), 38.7 (CH), 50.7 (CH_2_), 51.2 (CH_2_), 53.0 (CH_2_), 56.5 (CH_2_); FTIR 3410, 2920, 2743, 1635, 1405, 1303, 1125 cm^−1^.

### 3.5. Procedure for Cyclopolymerization of ***7*** with V-50

A 10 mL test tube was charged with **7** (0.47 g, 1.59 mmol), distilled water (1 mL), V-50 initiator (9 mg, 33.2 μmol). The tube was sealed with a septum and the solution was purged with nitrogen gas for ten minutes. The mixture was stirred and heated at 90 °C for four days. The solvent was removed under reduced pressure and the solid residue was dissolved in distilled water and dialyzed for three days (MWCO 1000, against distilled water). The solvent was removed under reduced pressure to yield polymer **9** (24 mg, 5%). Mp decomposed above 220 °C; ^1^H-NMR (300 MHz, D_2_O) δ 1.1 (br s), 2.6 (br s), 2.7 (br s), 2.9 (br s), 3.1(br s), 3.2 (br s), 3.4 (br s), 3.6 (br s), 8.5 (s, 1H), 8.52 (s, 1H); ^13^C-NMR (75 MHz, D_2_O) δ 27.2 (CH_2_), 29.1 (CH_2_), 42.4 (CH), 43.0 (CH_2_), 43.3 (CH_2_), 56.6 (CH_2_), 57.0 (CH_2_), 60.4 (CH_2_), 120.4 (C), 147.2 (CH), 151.7 (C), 152.5 (C); UV-VIS λ_max_ 260 nm; IR (KBr) 3362, 2922, 2851, 1661, 1632, 1468, 1419 cm^−1^.

### 3.6. Procedure for Cyclopolymerization of ***7*** with Ammonium Persulfate

A 10 mL test tube was charged with **7** (257.7 mg, 0.82 mmol), distilled water (1 mL) and ammonium persulfate (3.68 mg, 16.5 μmol). The tube was sealed with a septum and the mixture was purged with nitrogen gas for ten minutes. The tube was heated at 90 °C with stirring for four days. The polymer phased out with the addition of acetone to yield a viscous-orange oily precipitate. The oily layer was separated, dissolved in distilled water and dialyzed for four days (MWCO 3500) against distilled water. The solvent was then evaporated under reduced pressure to yield polymer **9** (44 mg, 17%): Mp decomposed above 220 °C; ^1^H-NMR (300 MHz, D_2_O) δ 1.1 (br s), 2.6 (br s), 2.7 (br s), 2.89 (br s), 3.1 (br s), 3.2 (br s), 3.4 (br s), 3.6 (br s), 8.5 (s, 1H), 8.52 (s, 1H); ^13^C-NMR (75 MHz, D_2_O) δ 27.2 (CH_2_), 29.1 (CH_2_), 42.4 (CH), 43.0 (CH_2_), 43.3 (CH_2_), 56.6 (CH_2_), 57.0 (CH_2_), 60.4 (CH_2_), 120.4 (C), 147.2 (CH), 151.7 (C), 152.5 (C); UV-VIS: λ_max_ 260 nm; IR (KBr) 3362, 2922, 2851, 1661, 1632, 1468, 1419 cm^−1^.

### 3.7. Procedure for the Cyclo-Copolymerization of ***7*** with V-50

A glass tube (8 mm i.d., 11 mm o.d.) was charged with **7** (109.8 mg, 0.372 mmol), a solution of DMSO (1.138 mL) containing SO_2_ (24 mg, 0.372 mmol) and V-50 (15 mg, 55.3 μmol). The mixture was degassed (freeze-thawed) three times, frozen and the tube was sealed under reduced pressure, allowed to warm up to room temperature and placed in a preheated sand bath at 70 °C for three days. The polymer was precipitated with ethanol, filtered, washed repeatedly with ethanol and dried under reduced pressure to form polymer **8** (8.9 mg, 7%): Mp decomposed above 250 °C; ^1^H-NMR (300 MHz, D_2_O) δ 1.37 (br s), 3.0 (br s), 3.36 (br s), 3.58 (br s), 3.66 (br s), 8.47 (s, 1H), 8.49 (s, 1H); ^13^C-NMR (75 MHz, D_2_O) δ 35.4 (CH), 36.9 (CH), 43.0 (CH_2_), 53.9 (CH_2_), 54.1 (CH_2_), 56.8 (CH_2_), 59.4 (CH_2_), 120.7 (C), 147.2 (CH), 147.5 (CH), 152.2 (C), 151.7 (C); UV-VIS: λ_max_ 260 nm; IR (KBr) 3385, 1684, 1517, 1419, 1350, 1306, 1173, 1127 cm^−1^.

### 3.8. Procedure for Cyclo-Copolymerization of ***4*** with V-50

A glass tube (8 mm i.d., 11 mm o.d.) was charged with **4** (0.5 g, 1.94 mmol), a solution of methanol (3.76 mL) containing SO_2_ (0.124 g, 1.94 mmol) and V-50 (15 mg, 55.3 μmol). The mixture was degassed (freeze-thawed) three times, frozen and the tube was sealed under reduced pressure, allowed to warm up to room temperature and placed overnight in a preheated sand bath at 70 °C to yield a gelatinous orange precipitate. The solvent was decanted, the gelatinous precipitate was washed with MeOH, dissolved in aqueous HCl (10%) and dialyzed for one day (MWCO 3500) against distilled water, during which a gelatinous layer formed in the dialysis tube with the liquid layer on top. The top layer was decanted, the gelatinous layer was dissolved in aqueous HCl (10%) and the solvent was evaporated under reduced pressure to yield polymer **8** as a white solid (0.24 g, 35%). Mp decomposed above 250 °C; ^1^H-NMR (300 MHz, D_2_O) δ 1.3 (br s), 3.0 (br s), 3.3 (br s), 3.5 (br s), 3.6 (br s), 8.47 (s, 1H), 8.49 (s, 1H); ^13^C-NMR (75 MHz, D_2_O) δ 35.4 (CH), 36.9 (CH), 43.0 (CH_2_), 53.9 (CH_2_), 54.1 (CH_2_), 56.8 (CH_2_), 59.4 (CH_2_), 120.7 (C), 147.2 (CH), 147.5 (CH), 152.2 (C), 151.7 (s); UV-VIS λ_max_ 260 nm; IR (KBr) 3385, 1684, 1517, 1419, 1350, 1306, 1173, 1127 cm^−1^.

## 4. Conclusions

The synthesis of 9-(2-diallylaminoethyl)adenine hydrochloride salt from adenine in four steps was reported. The free-radical homopolymerization and copolymerization of this salt with sulfur dioxide exhibited relatively low degree of polymerization under the conditions investigated in this study. In contrast, the cyclo-coplymerization of 9-(2-diallylaminoethyl)adenine with SO_2_ resulted in novel low molecular weight oligomers in good yields. Further studies are currently underway to determine if related purine and pyrimidine-substituted 2-diallylaminoethyl derivatives display similar free-radical polymerizations.
